# Low serum albumin and the acute phase response predict low serum selenium in HIV-1 infected women

**DOI:** 10.1186/1471-2334-6-85

**Published:** 2006-05-19

**Authors:** Paul K Drain, Jared M Baeten, Julie Overbaugh, Mark H Wener, Daniel D Bankson, Ludo Lavreys, Kishorchandra Mandaliya, Jeckoniah O Ndinya-Achola, R Scott McClelland

**Affiliations:** 1 School of Medicine, University of Washington, 1959 NE Pacific, A-300 Health Sciences, Box 356340, Seattle, WA 98105, USA; 2 Department of Medicine, University of Washington, 1959 NE Pacific, A-300 Health Sciences, Box 356340, Seattle, WA 98105, USA; 3 Department of Laboratory Medicine, University of Washington, 1959 NE Pacific, A-300 Health Sciences, Box 356340, Seattle, WA 98105, USA; 4 Clinical Nutrition Research Unit Laboratory Core, University of Washington, 1959 NE Pacific, A-300 Health Sciences, Box 356340, Seattle, WA 98105, USA; 5 Department of Epidemiology, University of Washington, 1959 NE Pacific, A-300 Health Sciences, Box 356340, Seattle, WA 98105, USA; 6 Veterans Affairs Puget Sound Health Care System, Seattle, USA; 7 Divisions of Human Biology and Public Health Sciences, Fred Hutchinson Cancer Research Center, Seattle, USA; 8 Department of Pathology, Coast Provincial General Hospital, Mombasa, Kenya; 9 Department of Medical Microbiology, University of Nairobi, Nairobi, Kenya

## Abstract

**Background:**

Low serum selenium has been associated with lower CD4 counts and greater mortality among HIV-1-seropositive individuals, but most studies have not controlled for serum albumin and the presence of an acute phase response.

**Methods:**

A cross-sectional study was conducted to evaluate relationships between serum selenium concentrations and CD4 count, plasma viral load, serum albumin, and acute phase response markers among 400 HIV-1-seropositive women.

**Results:**

In univariate analyses, lower CD4 count, higher plasma viral load, lower albumin, and the presence of an acute phase response were each significantly associated with lower serum selenium concentrations. In multivariate analyses including all four of these covariates, only albumin remained significantly associated with serum selenium. For each 0.1 g/dl increase in serum albumin, serum selenium increased by 0.8 μg/l (p < 0.001). Women with an acute phase response also had lower serum selenium (by 5.6 μg/l, p = 0.06).

**Conclusion:**

Serum selenium was independently associated with serum albumin, but not with CD4 count or plasma viral load, in HIV-1-seropositive women. Our findings suggest that associations between lower serum selenium, lower CD4 count, and higher plasma viral load may be related to the frequent occurrence of low serum albumin and the acute phase response among individuals with more advanced HIV-1 infection.

## Background

Nutritional deficiencies have long been recognized as an important problem among HIV-1-seropositive individuals, particularly in resource-limited settings [1]. Micronutrient deficiencies have been associated with more rapid HIV-disease progression and higher HIV-1 related mortality [2,3]. In some studies, micronutrient supplementation has delayed time to AIDS and improved survival, suggesting that supplementation could offer a simple and relatively inexpensive strategy to slow HIV-1 progression [4,5].

Selenium is an antioxidant micronutrient that is an essential element of selenoproteins, including selenoprotein P and glutathione peroxidase. Among HIV-1-seropositive individuals, lower serum selenium concentrations have been associated with lower CD4 counts, more advanced HIV-1 disease, and greater HIV-1 related mortality [6-10]. However, most studies have not controlled for low serum albumin, which binds non-specifically to selenium in serum, or for the presence of an acute phase response, which alters hepatic production of albumin and other serum proteins [11,12]. We sought to determine whether serum selenium was independently associated with CD4 count or plasma viral load after adjusting for serum albumin and the presence of an acute phase response.

## Methods

### Study design

A cross-sectional study was conducted using baseline data from 400 HIV-1-seropositive women enrolled in a randomized trial of micronutrient supplementation [13]. Data were collected between September 1998 and June 2000 at Coast Provincial General Hospital in Mombasa, Kenya. Women between 18 and 45 years old were enrolled if they were not currently or recently (last 3 months) pregnant, taking vitamin supplements, or using oral contraceptives. The enrollment criteria were based on the parent trial of micronutrient supplementation [13]. All participants were antiretroviral naïve. The protocol was approved by the institutional review boards of the University of Nairobi and the University of Washington, and all women provided written informed consent.

Detailed procedures and sample collection techniques have been previously described [13]. In brief, women were interviewed regarding demographic, sexual, and medical characteristics using a standardized questionnaire. A physical examination was performed. Blood was collected for lymphocyte subset analysis, quantitation of plasma HIV-1 RNA, and nutritional assays.

### Laboratory methods

Serum samples were protected from light, separated within 4 hours of collection, and stored at -70°C. Serological testing for HIV-1 was performed using an ELISA (Detect HIV 1/2, BioChem Immunosystems, Montreal, Canada), and confirmed with a second ELISA (Recombigen, Cambridge Biotech, Worcester, USA). Absolute CD4 counts were determined using a semiautomated system (Zymmune CD4/CD8 Cell Monitoring Kit, Bartels Inc., Issaquah, USA), which had a lower quantitation limit of 25 cells/μl. The quantity of HIV-1 RNA in plasma was determined using the Gen-Probe HIV-1 viral load assay (Gen-Probe Incorporated, San Diego, USA). The lower limit of quantification for the assay was 3 copies/reaction, which was equivalent to 12 copies/ml for the plasma volumes tested [14]. Serum selenium was quantified using graphite furnace atomic absorption spectrophotometry [15]. Serum albumin, C-reactive protein (CRP), and α_1_-acid glycoprotein (AGP) were determined by nephelometry (Dade Behring, Marburg, Germany).

### Statistical analysis

Data were analyzed using SPSS 12.0 (SPSS Inc., Chicago, USA). Low serum selenium was defined as a serum level ≤ 85 μg/l [16], a threshold that has been associated with adverse outcomes in HIV-1 infection [7,8]. An acute phase response was considered to be present if a participant had CRP ≥ 1 mg/dl [12] or AGP ≥ 100 mg/dl [17]. Univariate comparisons were performed using chi-square tests for dichotomous outcomes and t-tests for continuous outcomes. Multivariate comparisons were conducted using logistic and linear regression. Plasma HIV-1 RNA levels were log_10 _transformed to approximate a normal distribution.

## Results

### Study population

Baseline characteristics of this study population have been described [13]. In brief, participants had a mean age of 29 years [standard deviation (SD) ± 6] with 7 years (SD ± 4) of education. Participants were generally of low socioeconomic status, as evidenced by only 35 (9%) having a toilet in the home. Two hundred twenty (55%) participants were married. The women had a mean of 3 children (SD ± 2), and 73 (18%) were using injectable progesterone contraception (depot medroxyprogesterone acetate). The mean serum selenium concentration was 100 μg/l (SD ± 26).

### Comparison of women with low vs. normal serum selenium

Women with low serum selenium had more advanced immunosuppression, higher plasma viral loads, lower albumin, more frequent symptoms and signs of HIV-1 infection, and were more likely to have an acute phase response compared to women with normal serum selenium (Table 1). In a multivariate model including CD4 count, plasma viral load, albumin, and the acute phase response, only serum albumin concentration and the presence of an acute phase response remained significantly associated with low serum selenium. When included in the multivariate model, signs and symptoms of HIV-1 and body mass index were not independently associated with serum selenium and their inclusion did not significantly affect the association between selenium and albumin or the acute phase response, so these covariates were not included in the final multivariate model.

**Table 1:** Comparison of HIV-1-Seropositive Women with Low (≤ 85 μg/l) versus Normal (>85 μg/l) Serum Selenium Concentrations.

	Mean (± SD) or Number (%)			Multivariate Logistic Regression^1^	
		
	Low serum selenium (n = 104)	Normal serum selenium (n = 296)	p-value^2^	Adjusted Odds Ratio (95% CI)	p-value
CD4 count (cells/μl)	216 (± 182)	300 (± 210)	<0.001	0.90 (0.77, 1.06)^3^	0.2
Plasma HIV-1 RNA (log_10 _of copies/ml)	5.6 (± 0.9)	5.3 (± 1.0)	0.01	0.84 (0.61, 1.17)	0.3
Serum albumin (g/dl)	2.86 (± 0.75)	3.34 (± 0.68)	<0.001	0.93 (0.90, 0.97)	<0.001
Acute phase response^4^	73 (70%)	129 (44%)	<0.001	1.83 (1.05, 3.18)	0.03
HIV-1 symptoms^5^	83 (80%)	198 (67%)	0.008	-	-
HIV-1 signs^6^	34 (33%)	57 (19%)	0.01	-	-
Body mass index (kg/m^2^)	21.5 (± 3.5)	22.3 (± 4.7)	0.08	-	-

SD – standard deviation; CI – confidence interval

^1 ^CD4 count, plasma HIV-1 RNA, serum albumin were modeled as continuous variables, and the acute phase response was modeled as a dichotomous variable.

^2 ^Calculated by using t-tests for continuous variables and χ^2 ^tests for dichotomous variables.

^3 ^Odds ratio is per 100 CD4 cells/μl increase.

^4 ^The presence of C-reactive protein ≥ 1 mg/dl and/or α_1_-acid glycoprotein ≥ 100 mg/dl.

^5 ^Defined as fever for ≥ 1 month, diarrhea for ≥ 1 month, cough for ≥ 1 month, unintended weight loss of ≥ 5 kg during previous year, or itching skin rash during previous year.

^6 ^Defined as the presence of oral thrush, oral hairy leukoplakia, oral ulcer, maculopapular rash, or Kaposi's sarcoma.

### Correlates of serum selenium

In univariate analyses, higher CD4 count and higher serum albumin concentrations were associated with higher serum selenium concentrations, while higher plasma viral load, the presence of an acute phase response, and symptoms or signs of HIV-1 disease were associated with lower selenium concentrations (Table 2). In a multivariate model including CD4 count, plasma viral load, albumin, and the acute phase response, only albumin was significantly associated with serum selenium. Each 0.1 g/dl increase in serum albumin was associated with an 0.8 μg/l [95% confidence interval (CI) 0.4–1.2] increase in serum selenium. Women with an acute phase response had lower serum selenium concentrations than women without an acute phase response, although this association did not reach statistical significance. Signs or symptoms of HIV-1 and body mass index were not associated with serum selenium and did not substantially affect the associations between selenium and albumin or the acute phase response results, so these variables were not included in the final multivariate model. In separate multivariate models evaluating CRP and AGP as continuous covariates, neither of these inflammatory markers was independently associated with serum selenium (data not shown).

**Table 2:** Correlates of Serum Selenium Concentration (μg/l) among 400 HIV-1-Seropositive Women.

	Univariate Linear Regression		Multivariate Linear Regression	
		
	Coefficient (95% CI)	p-value	Coefficient (95% CI)	p-value
CD4 count (per 100 cells/μl increase)	1.8 (0.6, 3.0)	0.004	0.2 (-1.3, 1.7)	0.8
Plasma HIV-1 RNA (per 1 log_10 _copies/ml increase)	-3.5 (-6.1, -0.8)	0.01	0.6 (-2.7, 3.8)	0.7
Serum albumin (per 0.1 g/dl increase)	1.0 (0.7, 1.3)	<0.001	0.8 (0.4, 1.2)	<0.001
Acute phase response^1^	-10.8 (-15.8, -6.0)	<0.001	-5.4 (-10.9, 0.1)	0.06
HIV-1 symptoms^2^	-9.5 (-14.9, -4.0)	0.001	-	-
HIV-1 signs^3^	-7.0 (-13.0, -1.1)	0.02	-	-
Body mass index (kg/m^2^)	0.17 (-0.4, 0.7)	0.5	-	-

CI – confidence interval

^1^The presence of C-reactive protein ≥ 1 mg/dl and/or α_1_-acid glycoprotein ≥ 100 mg/dl.

^2 ^Defined as fever for ≥ 1 month, diarrhea for ≥ 1 month, cough for ≥ 1 month, unintended weight loss of ≥ 5 kg during previous year, or itching skin rash during previous year.

^3 ^Defined as the presence of oral thrush, oral hairy leukoplakia, oral ulcer, maculopapular rash, or Kaposi's sarcoma.

## Discussion

In this cross-sectional study of HIV-1-seropositive women, low serum selenium was independently associated with serum albumin and with the acute phase response, but not with CD4 count or plasma viral load. Further prospective studies may help determine whether associations between low serum selenium and low CD4 count [6,9] and more advanced HIV-1 disease [10] could be related to the frequent occurrence of hypoalbuminemia and the acute phase response in people with advanced HIV-1 infection.

Several ingested forms of selenium, including selenomethionine, bind non-specifically to albumin for transport to the liver [11,18-21]. The liver converts these compounds into selenocysteine, which is used to form various selenoproteins. In total, approximately 55% of selenium in human serum exists in selenoprotein P, another 17–32% exists bound to albumin, mostly in the form of selenomethionine, and only 10% of serum selenium is not protein bound [11,18,21]. Since low serum albumin has been independently associated with faster HIV-1 disease progression and higher mortality, low serum selenium may simply reflect a decline in serum albumin among people with more active or advanced HIV-1 disease [6,23].

The presence of an acute phase response is typically associated with a decrease of serum albumin and other plasma proteins [12]. Among HIV-1-seropositive individuals, the acute phase response has also been associated with low serum selenium and with HIV-1 disease progression and mortality [6,24]. One study found that CRP predicts mortality in HIV-1-infected women independent of serum albumin [25]. Our results suggest that the observed univariate associations between serum selenium and the acute phase response may have been due, at least in part, to decreased hepatic production of albumin and other plasma proteins in HIV-1-seropositive individuals with an acute phase response [18,22]. There may also be a redistribution of selenium from serum and liver to muscle tissue during an acute phase response [22].

Our study builds on previous analyses by examining the relationship between serum selenium concentrations, CD4 count, and plasma HIV-1 viral load in a large cohort of untreated HIV-1-seropositive adults. The size of this study enhanced our ability to conduct detailed multivariate analyses, which demonstrate the lack of a significant independent association between selenium and CD4 cell count or plasma viral load.

We have previously published the results of the micronutrient supplementation trial in which these women received six weeks of either a supplement containing B vitamins, vitamin C, vitamin E, and selenium or an identical placebo [13]. Following supplementation, women who received the supplement had slightly higher CD4 counts compared to those who received placebo, an effect that was also observed in a trial of an otherwise identical supplement that did not contain selenium [5]. It is not possible to disentangle the independent effects of selenium from the known effects of those other micronutrients that were provided in the same supplement. Thus, we were unable to use those longitudinal data to evaluate the associations between selenium supplementation and albumin, CD4 count, and plasma viral load.

The findings presented here should be interpreted in the context of the limitations of this study. Although cross-sectional studies are useful to define associations, it is not possible to infer with certainty that low albumin or the acute phase response were the cause of low measured serum selenium, although this relationship seems plausible because a large proportion of serum selenium is protein bound [18,22]. Regardless of the mechanism, the confounding bias demonstrated by our analyses was strong enough to nullify highly significant univariate associations between serum selenium and CD4 count and plasma viral load. However, these data cannot rule out the possibility that low serum selenium or a low antioxidant status was the cause of low serum albumin. Furthermore, because hypoalbuminemia may influence the relationship between serum selenium and total body selenium status, the measured serum selenium may not accurately reflect total body selenium in advanced HIV-1 infection. Data on dietary selenium intake were not collected in this population. Finally, because this study included only women, these results may not be generalizable to HIV-1-seropositive men.

The finding that serum selenium is not independently associated with CD4 count or plasma viral load may help to explain the results of small randomized and non-randomized trials of selenium supplementation among HIV-1-seropositive individuals. While one study found an increase in CD4/CD8 ratio after 12 weeks [26], none have demonstrated significant effects on the absolute CD4 cell count or plasma viral load [10,26,27]. However, a beneficial effect of selenium supplementation that is independent of CD4 count and plasma viral load is possible. In one randomized trial, selenium supplementation decreased hospital admissions due to infections among HIV-1 infected adults [28]. The trial did not report changes in biological markers of HIV-1 disease progression or the effect on HIV-1-related mortality.

## Conclusion

The results of this investigation demonstrate that serum selenium was not independently associated with CD4 count or plasma viral load among HIV-1-seropositive women. These findings indicate that studies assessing the impact of selenium on HIV-1 surrogate markers, such as CD4 cell count and plasma viral load, need to control for serum albumin levels and the presence of an acute phase response.

## Abbreviations

AGP – α_1_-acid glycoprotein

CI – Confidence Interval

CRP – C-reactive protein

SD – Standard Deviation

## Competing interests

The author(s) declare that they have no competing interests.

## Authors' contributions

PKD, JMB, KM, JON, and RSM designed the study. LL, JMB, KM and RSM collected data and provided study oversight. PKD and RSM analyzed data. PKD, JMB, JO, MHW, DDB, and RSM interpreted the results. PKD and RSM primarily wrote the manuscript. JMB, JO, MHW, DDH provided valuable insight for revising the manuscript. All authors read and approved the final manuscript.

## Pre-publication history

The pre-publication history for this paper can be accessed here:


